# Development and validation of four ferroptosis-related gene signatures and their correlations with immune implication in hepatocellular carcinoma

**DOI:** 10.3389/fimmu.2022.1028054

**Published:** 2022-10-11

**Authors:** Ying Zhang, He Ren, Chunting Zhang, Haihua Li, Qingzhi Guo, Haitao Xu, Lina Cui

**Affiliations:** ^1^ Department of Breast Surgery, Harbin Medical University Cancer Hospital, Harbin, China; ^2^ Department of General Surgery, Tianjin Medical University General Hospital, Tianjin Medical University, Tianjin, China; ^3^ Department of Nursing, Air Force Medical Center, People’s Liberation Army (PLA), Beijing, China; ^4^ Pediatric Nursing Station of Qitaihe Maternal and Child Health Hospital, Qitaihe, Heilongjiang Qitaihe Province, China; ^5^ Department of Renal Sixth, Heilongjiang Academy of Traditional Chinese Medicine, Harbin, Heilongjiang Harbin Province, China; ^6^ Department of Hepatobiliary and Pancreatic Surgery, The Affiliated Cancer Hospital of Harbin Medical University, Harbin, China

**Keywords:** ferroptosis, hepatocellular carcinoma, prognosis, tumor-infiltrating immune cells, immune checkpoint

## Abstract

Hepatocellular carcinoma (HCC) is one of the most common malignant tumors. This tumor presents with an insidious onset, rapid progression, and frequent recurrence. Ferroptosis is a newly discovered mode of programmed cell death that may play a key role in the progression of HCC. This study aimed to investigate the prognostic value of ferroptosis-related genes (FRGs) in HCC and their impact on tumor immune function, thereby providing new insights into targeted therapy for HCC. First, 43 differentially expressed FRGs were identified using the TCGA database, and four prognostically relevant methylation-driven FRGs (*G6PD*, *HELLS*, *RRM2*, and *STMN1*) were screened *via* survival and methylation analyses. Gene co-expression, mutation, and clinicopathological characterization indicated that these four pivotal FRGs play essential roles in tumor progression. We also validated these four genes using transcriptomic and proteomic data as well as cohort samples from our patients. Moreover, receiver operator characteristic (ROC) curves confirmed that the signatures of the four FRGs were independent prognostic factors in HCC. Gene set enrichment analysis of the four FRGs showed statistically significant associations with pathways related to HCC proliferation. Finally, the TIMER and TISIDB databases indicated that the four FRGs were statistically significantly correlated with tumor-infiltrating immune cells and immune checkpoint expression. Taken together, this study provides information guiding a novel therapeutic strategy targeting FRGs for HCC treatment.

## Introduction

Primary liver cancer is one of the most common malignant tumors, and its incidence is increasing annually. Asia and Africa have the highest incidence of liver cancer worldwide, with a greater than 50% incidence in China (1, 2). Most liver cancers occur following liver fibrosis or cirrhosis. Chronic infection with the hepatitis B or hepatitis C viruses are the main cause of these diseases; cirrhosis may also be caused by other risk factors, including alcohol consumption, alcoholic fatty liver associated with metabolic syndrome, and non-alcoholic fatty liver disease (3, 4).

Hepatocellular carcinoma (HCC) accounts for approximately 70%-90% of primary liver tumors and is the most common primary liver cancer. The first choice for HCC treatment is surgical resection and liver transplantation; however, patients often have to wait for a long time due to the shortage of donor livers and limitations in regard to immune matching (5). Second, sorafenib, which was approved for clinical use in 2007, represents a major advancement in drug therapy for HCC. However, its phase III clinical trial only extended the average survival time of patients from 7.9 months to 10.7 months (6). Therefore, it is particularly important to identify specific early diagnostic markers for HCC and to develop novel therapeutic measures for effective treatment.

The widespread use of next-generation sequencing and other gene microarray technologies has led to the creation of a large number of public tumor databases, and the integration and reanalysis of this data can provide valuable clues regarding the pathogenesis of malignancies. Ferroptosis is an iron-dependent form of programmed cell death induced by lipid peroxide damage (mainly in mitochondria) (7). During tumorigenesis, ferroptosis can regulate tumor development by relying on the release of damage-associated molecular patterns (DAMPs) in the tumor microenvironment and the activation of immune responses triggered by ferroptosis (8). Ferroptosis plays a crucial role in HCC development (9). A recent study reported that Rb may show beneficial effects in reference to sorafenib resistance by targeting iron death (10). In addition, it was also reported that CISD1 negatively regulates ferroptosis in HCC (11). However, the impact of ferroptosis-related genes (FRG) on the prognosis of HCC and immune regulation is not yet clear. In this study, we constructed four FRG prognostic signatures using The Cancer Genome Atlas (TCGA) database and validated the model using the Gene Expression Omnibus (GEO) cohort. Moreover, we validated mRNA expression levels in HCC samples and explored their potential mechanisms in HCC as well as their relationships with immune functions.

## Materials and methods

### Data acquisition

HCC transcriptome data from The Cancer Genome Atlas (TCGA; http://www.cancergenome.nih.gov/) were downloaded as an analytic dataset. HCC data from the International Cancer Genome Consortium (ICGC) and five original HCC-associated gene expression datasets, including GSE22058, GSE14250, GSE54236, GSE64014, and GSE63898, were downloaded from the NCBI GEO database (https://www.ncbi.nlm.nih.gov/nih.g/gds) as an external validation cohort. Ferroptosis-related genes were downloaded from the FerrDb database (http://www.zhounan.org/ferrdb/index.html), including 123 ferroptosis marker genes, 150 ferroptosis driver genes, and 109 ferroptosis suppressor genes. In total, 259 ferroptosis-related genes were obtained for analysis following the removal of duplicate genes from the three subgroups of the ferroptosis genome.

### Differentially expressed FRGs: acquisition and functional analysis

According to the employed filtering criteria (|log_2_FC|>1.5 and an adjusted P-value of <0.05), differentially expressed genes in liver tumors and adjacent normal tissues were analyzed in the TCGA cohort using the “limma” package in R (The R Project for Statistical Computing, Vienna, Austria) and were intersected with FRGs to obtain differentially expressed genes for ferroptosis. To explore the potential biological functions of FRGs and related pathways, gene ontology (GO) enrichment and Kyoto Encyclopedia of Genes and Genomes (KEGG) pathway analysis were performed using the DAVID database (12) (https://david.ncifcrf.gov/).

### Prognostic value of ferroptosis-related DEGs

We further identified the ferroptosis-related DEGs related to overall survival (OS) and relapse-free survival (RFS) using univariate Cox regression analysis. The “survival” package in R was used in the process of analysis, and a P-value of <0.05 was adopted as the screening criterion.

### UALCAN and human protein atlas database analysis

The University of Alabama at Birmingham cancer data analysis (UALCAN) portal is a comprehensive, user-friendly, and interactive web resource built using Perl-CGI programming that generates high-quality graphics for analyzing cancer omics data (13). This database was used to evaluate the epigenetic regulation of expression for 11 FRGs *via* promoter methylation; four hub genes were selected from the 11 FRGs for analysis of protein expression levels and various clinical disease characteristics. The Human Protein Atlas (HPA) is a Swedish-based platform initiated in 2003 for the purpose of mapping all human proteins in cells, tissues, and organs through the integration of various omics technologies, including antibody-based imaging, mass spectrometry-based proteomics, transcriptomics, and systems biology. Protein expression data for four FRGs was obtained from the HPA database.

### cBioPortal and GeneMANIA analysis

The cBio Cancer Genomics Portal (cBioPortal) is a tool that integrates visualization of somatic mutations as well as data on DNA copy number alterations, mRNA and microRNA expression, DNA methylation, protein abundance, and phosphoprotein abundance (14). The frequency of genetic alterations in the four FRGs in patients with HCC and their associations with survival outcomes were investigated using cBioPortal. The GeneMANIA database (https://www.genemania.org) was used to construct four FRGs and 30 related gene interaction networks (15).

### Collection and validation of clinical specimens

Ten tumor and peri-tumor tissues collected from HCC patients at the Tianjin Medical University General Hospital between October 2015 and July 2017 were selected from evaluation in the present study. TRIzol reagent (Invitrogen, Waltham, MA, USA) was used to extract total RNA from the tissue; this was then reverse-transcribed into complementary DNA (cDNA) for quantitative revere transcription polymerase chain reaction (qRT-PCR). β-actin was used as an internal control, and the relative expression level was determined according to the fold change (2-^ΔΔCT^). The primer sequences are shown in **Table S1**. This study was approved by the ethics committee of the Tianjin Medical University General Hospital. All patients provided their informed consent before participating in the study.

### Construction and validation of a prognostic signature with FRGs

We further evaluated the prognostic value of the candidate FRGs. The HCC dataset from the TCGA database and the GSE14520 dataset from the GEO database were employed to construct a predictive model based on ferroptosis-related prognostic characteristics using the Sangerbox tool. The integrated prognostic receiver operating characteristic (ROC) and survival curve tools for evaluating the relationship between risk scores and gene expression data, developed using the ggplot2 package in R, were used to perform multivariate Cox regression analysis for the four ferroptosis-related gene prognostic risk models. The median risk scores were used to classify patients into high- and low-risk groups.

### Immune infiltration analysis

The TIMER database (https://timer.cistrome.org/) is a comprehensive resource for the systematic analysis of six immune infiltrate cells (B cells, CD4+ T cells, CD8+ T cells, neutrophils, macrophages, and dendritic cells) across diverse cancer types (16). This database was used to investigate the correlation between the expression of four selected FRGs and tumor immune infiltration abundance (B cells, CD4+ T cells, CD8+ T cells, neutrophils, macrophages, and dendritic cells), tumor purity, and genetic copy number variation (CNV). In addition, immune, stromal, and ESTIMATE scores were calculated using the ESTIMATE algorithm (17).

### Relationship between pivotal FRGs and immune checkpoints in HCC

TISIDB (https://cis.hku/hk/TISIDB/) is a web portal for tumor and immune system interactions that integrates multiple heterogeneous data types (18). The expression correlations between the four FRGs and immune checkpoints in HCC were evaluated using the TISIDB database. R >0.1 and P <0.05 were established as the selection criteria for determining statistical significance.

### Gene set enrichment analysis

Gene Set Enrichment Analysis (GSEA) is a method that uses predefined gene sets to rank genes according to their differential expression in two types of samples and then tests whether a predefined set of genes is enriched at the top or bottom of this ranking table (19). The HCC RNA-seq data from the TCGA database were downloaded and subjected to GSEA for four hub FRGs. After performing the permutation test 1,000 times, gene sets generating a P-value of <0.05 were considered statistically significant.

### Statistical analysis

Statistical data were analyzed using GraphPad Prism software (version 8.0; San Diego, CA, USA). Differences between the two groups were compared using Student’s t-tests, and the results were expressed as means ± standard deviations. A P-value of <0.05 was considered the threshold for statistical significance.

## Results

### Identification of ferroptosis-related DEGs and enrichment analysis

**Figure 1:**
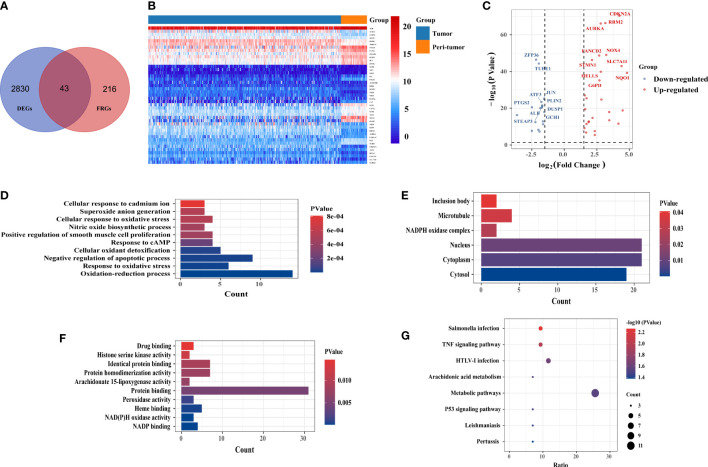
Identification of candidate ferroptosis-related differentially expressed genes (FR-DEGs) and enrichment analysis. **(A)** Venn plot of the 43 FR-DEGs in the TCGA-LIHC cohort. **(B)** the heat map showed the expression of 43 FR-DEGs in tumor and peri-tumor tissues. **(C)** Volcano plot of 43 FR-DEGs in TCGA-LIHC cohort. Blue dots: downregulated genes; Red dots: upregulated genes. **(D)** Biological processes (BP) terms. **(E)** Cellular components (CC) terms. **(F)** Molecular (MF) terms. **(G)** The Kyoto Encyclopedia of Genes and Genomes(KEGG) pathways.

A total of 2,873 DEGs were identified in the TCGA- liver hepatocellular carcinoma (LIHC) cohort, 43 of which were differentially expressed FRGs (**Figure 1A**); 17 genes were downregulated and 26 genes were upregulated (**Figure 1C**). The heatmap shows the distribution of the 43 FRGs (**Figure 1B**). GO and KEGG enrichment analyses were performed to elucidate the biological functions and pathways for 43 ferroptosis-related (FR)-DEGs. Regarding biological processes (BP), FR-DEGs were statistically significantly enriched within the following pathways: the oxidation-reduction process, response to oxidative stress, negative regulation of the apoptotic process, and cellular oxidant detoxification (**Figure 1D**). Cellular component (CC) analysis revealed that DEGs were mainly enriched in the cytosol, cytoplasm, nucleus, and nicotinamide adenine dinucleotide phosphate (NADPH) oxidase complex (**Figure 1E**). Regarding molecular function (MF), FR-DEGs were most strongly enriched in NADP binding, NAD(P)H oxidase activity, heme binding, and peroxidase activity pathways (**Figure 1F**). On KEGG pathway analysis, FRGs were notably associated with metabolic signaling pathways, tumor necrosis factor (TNF) signaling, arachidonic acid metabolism, and the p53 signaling pathway (**Figure 1G**).

### Selection of hub ferroptosis-related DEGs

**Figure 2:**
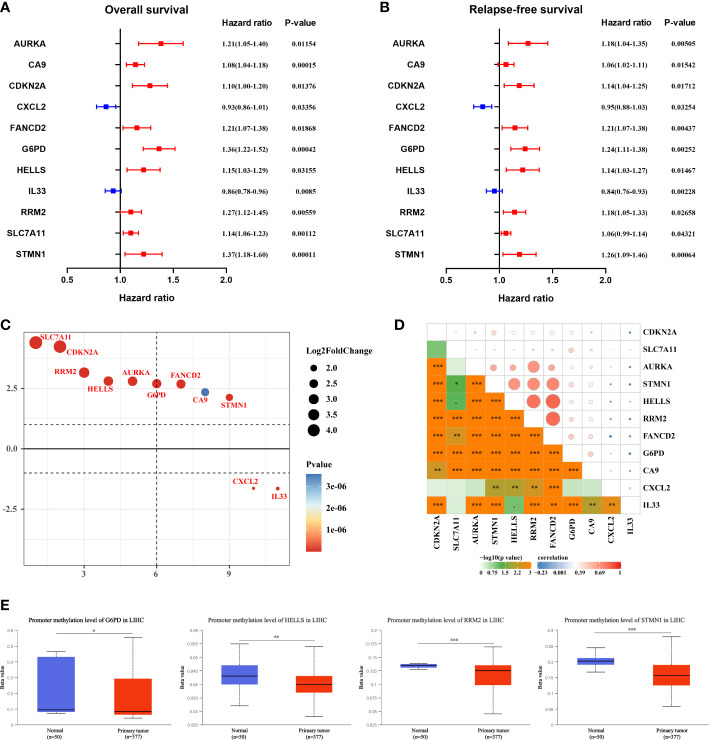
Selection of hub ferroptosis-related differentially expressed genes. **(A, B)** Forest plot for the univariate Cox regression analysis of OS and RFS with 11 FRGs. P-value <0.05 exhibited statistical significance. **(C)** The differential expression ranking of 11 hub FGRs. **(D)** Pearson’s correlation analysis of 11 FRGs in TCGA-LIHC. *P <0.05; **P <0.01; ***P <0.001. **(E)** The methylation levels of G6PD, HELLS, RRM2, and STMN1 in HCC and peri-tumor tissues were determined using the UALCAN database.

Univariate Cox regression analysis revealed that 11 FR-DEGs were statistically significantly associated with both OS and RFS (**Figures 2A, B**). Next, we ranked the differential expression of these 11 prognosis-related FRGs, as shown in **Figure 2C**. Moreover, we evaluated Pearson correlations between the 11 FRGs in the TCGA-LIHC cohort, with the results indicating statistically significant correlations between the FRGs (**Figure 2D**).

DNA methylation (DNAm) is an essential epigenetic process that can affect pre-transcriptional genetic imprinting, genomic stability, and cell fate. Subsequently, to elucidate the potential mechanism of aberrant regulation of the 11 FRGs in HCC tissues, methylation expression level analysis was performed using UALCAN. The results revealed that the mean methylation levels of *G6PD*, *HELLS*, *RRM2*, and *STMN1* were significantly lower in HCC tissues than in peri-tumor tissues (**Figure 2E**).

### Identification of ferroptosis-interacting genes and analysis of genetic alterations in HCC

**Figure 3:**
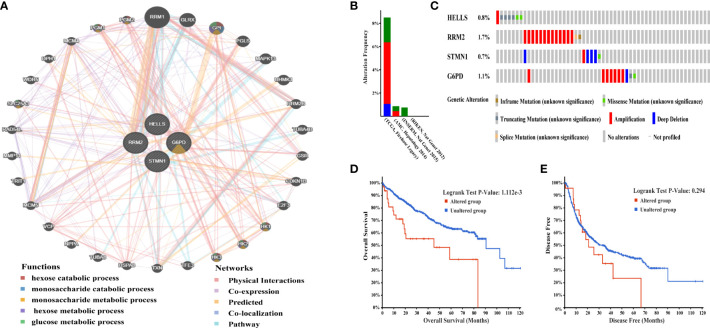
Interaction network and genetic alterations of four hub FRGs in HCC. **(A)** The gene-gene interaction network of four hub FRGs and 30 neighboring genes was constructed. **(B)** Alterations of the four FRGs in six datasets: (Liver Hepatocellular carcinoma, TCGA, Firehose Legacy; Liver Hepatocellular carcinoma, RIKEN, Nat Genet 2012; Liver Hepatocellular carcinoma, AMC, Hepatology 2014; Liver Hepatocellular Adenoma and Carcinomas, MSK, PLOS One 2018; Hepatocellular carcinomas, INSERM, Nat Genet 2015; Hepatocellular carcinomas, MSK, Clin Cancer Res 2018). **(C)** Alteration frequencies of 4 FRGs were based on the six datasets described earlier. **(D)** Kaplan-Meier plots comparing OS in cases with and without alterations 4 FRGs alterations. **(E)** Kaplan-Meier plots comparing DFS in cases with and without alterations 4 FRGs alterations.

Next, GeneMania was used to construct the gene-gene interaction network for the four evaluated methylation-driven FRGs and the altered neighboring genes. The results showed that the 30 most frequently altered genes were strongly associated with the four FRGs (**Figure 3A**). Functional analysis indicated that these genes were notably related to metabolism-related pathways (**Figure 3A**). The genetic alterations of the four evaluated FRGs in patients with HCC were analyzed using cBioPortal.

**Figure 4:**
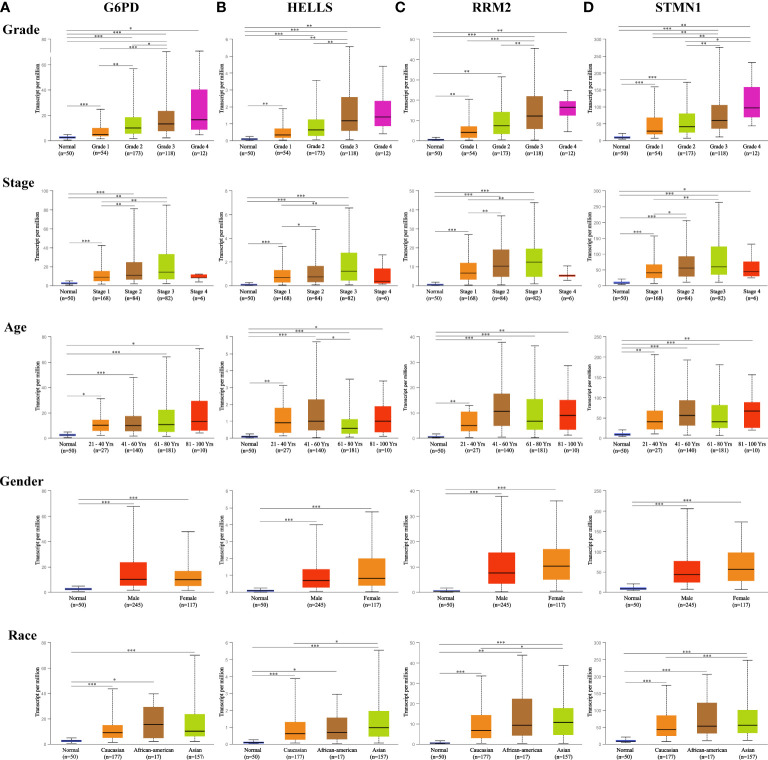
Expression of four hub FRGs in HCC stratified by clinical parameters in the UALCAN database. Boxplot showing the mRNA expression of four hub FRGs in HCC patients by stage, grade, age, sex, and race, respectively. **(A)** G6PD, **(B)** HELLS, **(C)** RRM2, **(D)** STMN1. *P <0.05; **P <0.01; ***P <0.00001.

A total of 1,022 patients were enrolled from within the following six HCC datasets: Liver Hepatocellular Carcinoma (TCGA, Firehose Legacy); Liver Hepatocellular Carcinoma, (RIKEN); Liver Hepatocellular Carcinoma (AMC); Liver Hepatocellular Adenoma and Carcinomas (MSK); Hepatocellular Carcinomas (INSERM); Hepatocellular Carcinomas (MSK). Genetic alterations of the four hub FRGs in the three datasets showed incidence rates of 8.49% (32/377), 0.87% (2/231), and 0.82% (2/243), respectively (**Figure 3B**). The four FRGs showed various genetic alterations, including in-frame mutations, missense mutations, truncating mutations, splice mutations, amplifications, and deep deletions (**Figure 3C**). Cases with four hub FRGs alterations were statistically significantly associated with a worse OS (P = 1.112e^-3^) in patients with HCC (**Figure 3D**). However, there was no statistical difference between the four hub FRG alterations and DFS (**Figure 3E**). Moreover, four FRGs were notably differentially expressed in HCC tissues in patients with different tumor grades, stages as well as differing characteristics in terms of age, gender, and race based on analysis conducted within the UALCAN dataset (**Figures 4A** **–D**).

### Validation of the expression levels of four FRGs

**Figure 5:**
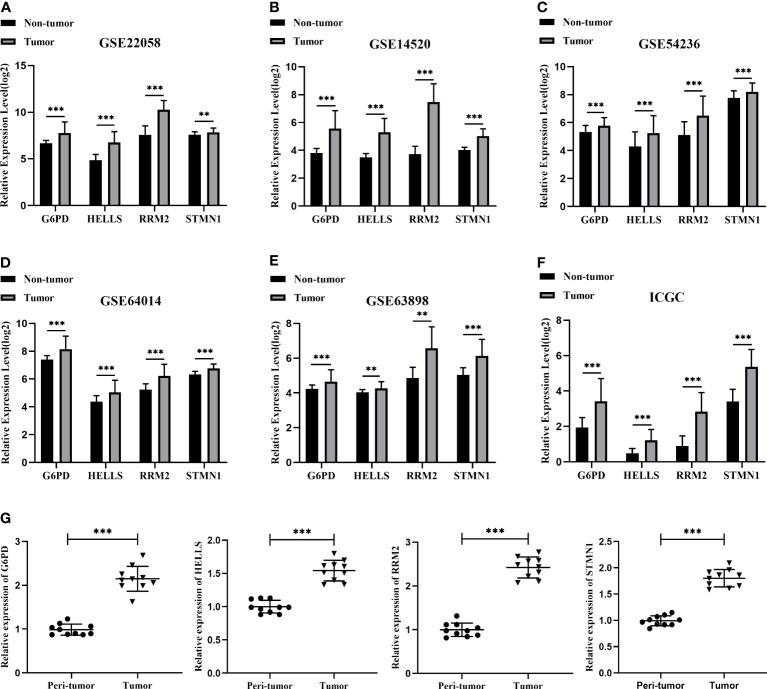
Validation of the mRNA expression for four hub FRGs. **(A)** Validation of four hub FRGs in GSE22058. **(B)** Validation of hub FRGs in GSE14520. **(C)** Validation of four hub FRGs in GSE54236. **(D)** Validation of four hub FRGs in GSE64014. **(E)** Validation of four hub FRGs in GSE63898. **(F)** Validation of four hub FRGs in ICGC. **(G)** Scatter plots of G6PD, HELLS, RRM2, and STMN1 were constructed using qRT-PCR data of our own patients’ cohort. (*P <0.05; **P <0.01; ***P <0.001).

**Figure 6:**
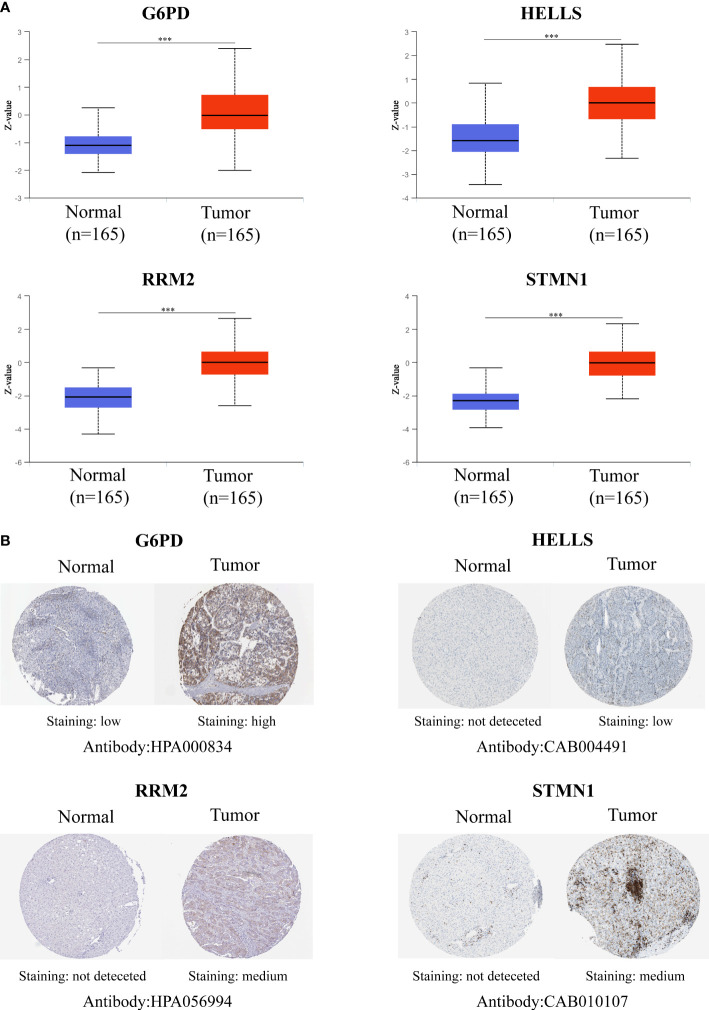
Validation of the Protein expression for four hub FRGs. **(A)** Protein expressions of four hub FRGs were significantly up-regulated in patients with HCC from the CPTAC database. (*P <0.05; **P <0.01; ***P <0.00001) **(B)** Representative immunohistochemistry staining of four hub FRGs. Protein expression levels of G6PD, HELLS, RRM2, and STMN1 in HCC tissue were obtained from the Human Protein Atlas.

To verify the transcript expression levels of the four evaluated hub FRGs, we downloaded the GSE22058, GSE14520, GSE54236, GSE64014, GSE63898, and ICGC datasets; we found that the transcript levels of the four FRGs showed the same tendencies as in the TCGA database, and similarly reached statistical significance (**Figures 5A** **–F**). In addition, the results of the bioinformatics analysis were validated using qRT-PCR conducted within 10 pairs of HCC and peri-tumor tissues. The results showed that G6PD, HELLS, RRM2, and STMN1 were highly upregulated in HCC tissues (**Figure 5G**), consistent with the bioinformatics findings. To validate the four FRGs at the protein level, protein expression data were obtained from the Clinical Proteomic Tumor Analysis Consortium (CPTAC) and Human Protein Atlas (HPA) databases. The protein expression levels of G6PD, HELLS, RRM2, and STMN1 showed a trend similar to that of the mRNA transcript levels (**Figures 6A, B**). All the above results indicate that four hub FRGs are overexpressed in HCC, in terms of both transcription and protein expression.

### Construction and validation of four prognostic signatures

**Figure 7:**
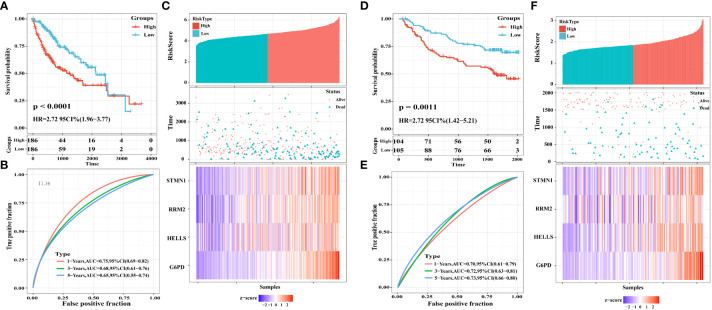
Construction and validation of a four-FRGs prognostic risk signature. **(A)** Distribution of the four-FRG signature in Kaplan-Meier survival curve for the TCGA-LIHC training set. **(B)** ROC curve and AUC for predicting OS. **(C)** TCGA-LIHC training set focused on risk score, survival time, survival status, and expression of the four-FRG signature. **(D)** Distribution of the four-FRG signature in the Kaplan-Meier survival curve for the GSE14520 validation set. **(E)** ROC curve and AUC for predicting OS. **(F)** TGSE14520 validation set focused on risk score, survival time, survival status, and expression of the four-FRG signature.

To explore the classification performance of the four FRG prognostic signatures, we used the TCGA cohort as a training dataset to construct a prognostic model. The Sangerbox tool was used to calculate the risk score for each sample and to classify TCGA cohort samples into high- and low-risk groups based on the median risk score. Consistently, Kaplan-Meier curves (**Figure 7A**) showed that OS was statistically significantly worse in the high-risk group of HCC patients (P <0.0001).

Moreover, we evaluated the predictive effect of prognostic characteristics on OS in HCC patients using time-dependent ROC curves, with the area under the curve (AUC) showing OS rates of 0.75, 0.68, and 0.65 at one, three, and five years, respectively; these results reflect the high sensitivity and specificity of the prognostic model (**Figure 7B**). Risk scores, survival time, survival status, and heat maps for the four FGRs are shown in **Figure 7C**.

To check the robustness of the four FRG models constructed from the TCGA cohort, the GSE14520 dataset from the GEO database was externally validated. Consistent with the results obtained from the TCGA cohort, Kaplan-Meier survival curves constructed using data from the GSE14520 validation cohort showed that HCC patients in the high-risk group had a statistically significantly lower OS, as shown in **Figure 7D** (P = 0.0011). The AUCs for the four FRG signatures were 0.70, 0.72, and 0.73 at one year, three years, and five years, respectively (**Figure 7E**). Risk scores, survival time, survival status, and expression heatmaps for the four FGRs are shown in **Figure 7F**.

### Investigation of statistically significant pathways for the four FRGs using GSEA

**Figure 8:**
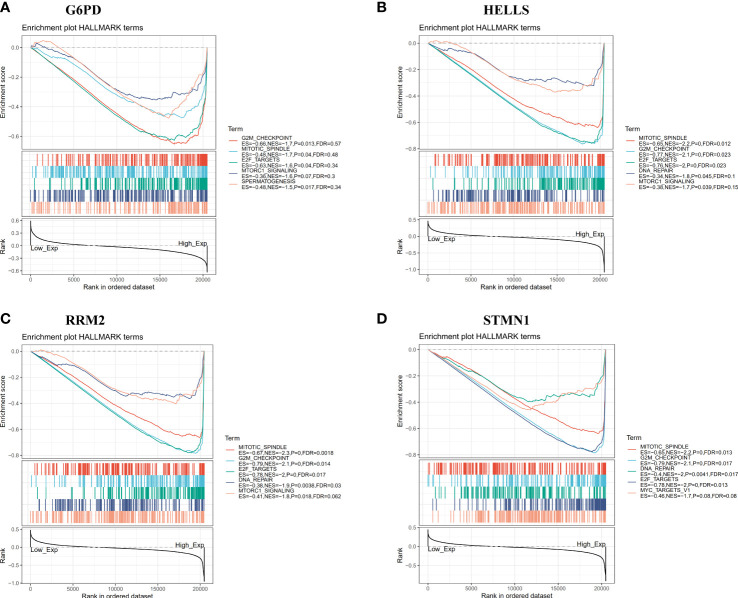
Gene set enrichment analysis (GSEA) of 4 hub FRGs in the TCGA-LIHC dataset. **(A–D)** Top five gene sets (according to GSEA enrichment score) for G6PD, HELLS, RRM2, and STMN1.

To further explore the potential functionality of G6PD, HELLS, RRM2, and STMN1 in HCC, GSEA was performed using TCGA-LIHC RNA-seq data. As shown in **Figures 8A–D**, G6PD, HELLS, RRM2, and STMN1 were each enriched in the “G2M checkpoint,” “mitotic spindle,” and “E2F target” pathways in the high expression groups. Meanwhile, the “DNA repair” gene set was enriched in the high expression groups for HELLS, RRM2, and STMN1, whereas “MTORC1 signaling” was enriched in the G6PD, HELLS, and RRM2 high-expression groups. In high-risk samples, these pathways in high-risk samples were closely associated with cellular metabolism and carcinogenesis.

### Correlation analysis: FRG expression and infiltrating immune cells

**Figure 9:**
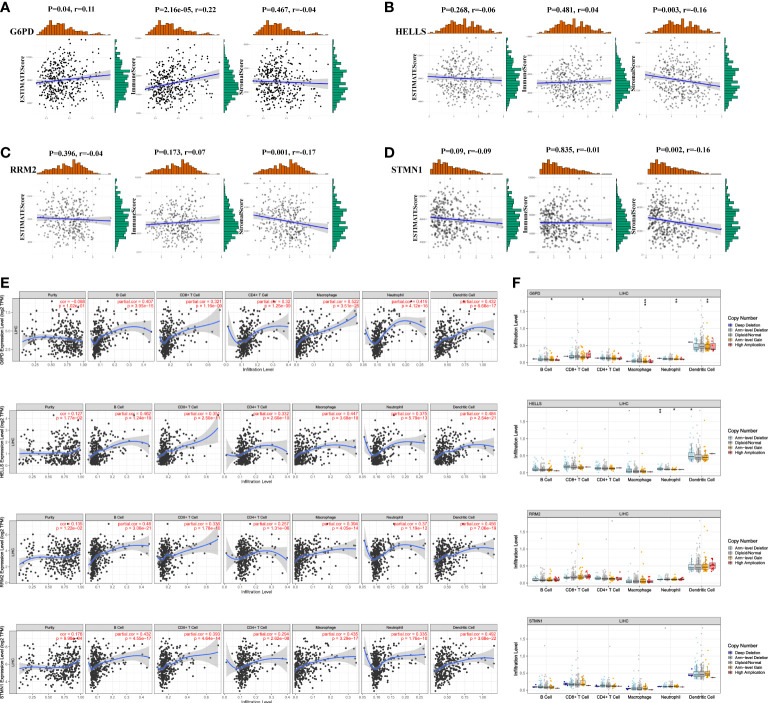
Association of four FRGs’ expression with immune infiltration level in HCC. **(A–D)** Association between immune/stromal/estimate score and four FRGs after ESTIMATE algorithm processed. **(E)** Correlation of FRGs including G6PD, HELLS, RRM2, and STMN1 with tumor purity and tumor infiltration immune cells. **(F)** The influence of copy number variation of G6PD, HELLS, RRM2, and STMN1 on the distribution of diverse infiltration immune cells. *P <0.05; **P <0.01; ***P <0.001.

We applied the ESTIMATE algorithm to conduct calculations informing a better understanding of the relationship between the estimated/immune/stromal scores and the evaluated FRGs. We found that G6PD was positively associated with immune scores (**Figure 9A**), and HELLS, RRM2, and STMN1 were negatively correlated with stromal scores (**Figures 9B** **–D**). Regarding the relationship between the four evaluated FRGs and tumor-infiltrating immune cells, the TIMER database was used to detect correlations between four prognostic FRGs and the abundance of immune cells. HELLS, RRM2, and STMN1 expression were positively associated with tumor purity (**Figure 9E**). Interestingly, strong associations were observed between the four FRGs and the infiltration of B cells, CD4+ T cells, CD8+ T cells, neutrophils, macrophages, and dendritic cells, with details shown in **Figure 9E**. Conversely, null or weak relationships were detected between CNV in the four hub FRGs and tumor immune-infiltrating cells (**Figure 9F**).

### Correlation between FRG expression immune checkpoints

**Figure 10:**
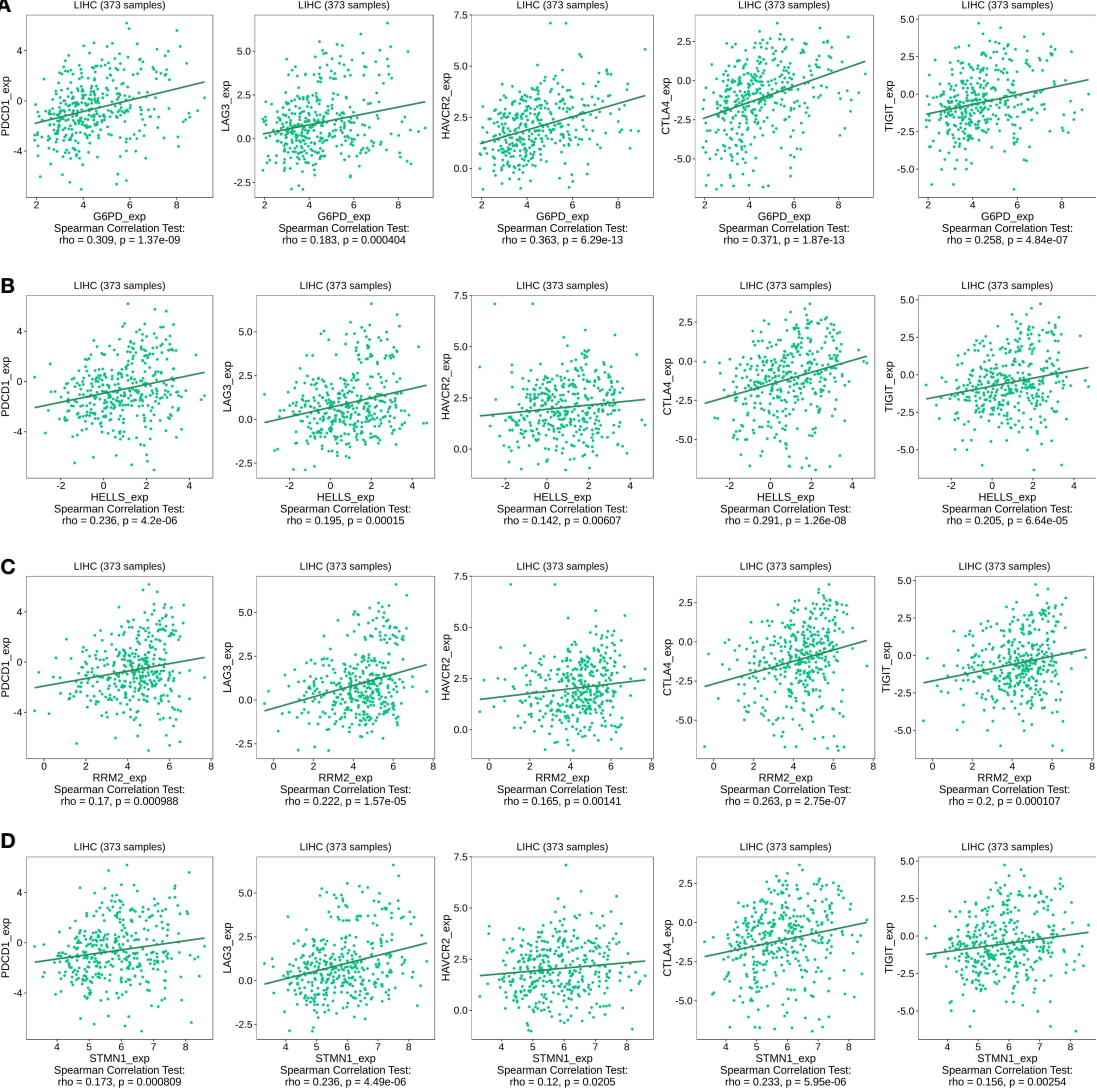
Correlation of four FRGs expression with immune checkpoint expression in HCC. **(A)** The expression correlation of G6PD with PDCD1, LAG3, HAVCR2, CTLA-4, and TIGIT expression in HCC was determined by the TISIDB database. **(B)** The expression correlation of HELLS with PDCD1, LAG3, HAVCR2, CTLA-4, and TIGIT expression in HCC was determined by the TISIDB database. **(C)** The expression correlation of RRM2 with PDCD1, LAG3, HAVCR2, CTLA-4, and TIGIT expression in HCC was determined by the TISIDB database. **(D)** The expression correlation of STMN1 with PDCD1, LAG3, HAVCR2, CTLA-4, and TIGIT expression in HCC was determined by the TISIDB database.

The abnormal activation of immune checkpoints is one mechanism through which cancer evades the immune system (20). Given the potential oncogenic effects of the four FRGs in HCC, we evaluated the associations between the four evaluated FRGs and PDCD1, LAG3, HAVCR2, CTLA-4, and TIGIT expression within the TISIDB database. As shown in **Figures 10A–D**, G6PD, HELLS, RRM2, and STMN1 expression was statistically significantly and positively correlated with PDCD1, LAG3, HAVCR2, CTLA-4, and TIGIT expression in HCC. These results suggest that overexpression of these four FRG-mediated immune checkpoints may be involved in liver tumor immune escape.

## Discussion

HCC is a clinically occurring malignant tumor with a high population incidence. Most patients are in the middle and late stages at diagnosis, with a complicated disease presentation and poor prognosis (21). Ferroptosis is defined as iron-ion-dependent programmed cell death, and the induction of cellular ferroptosis is an emerging approach in cancer therapy. Recently, several studies have demonstrated that promoting the sensitivity of cancer cells to ferroptosis contributes to the efficacy of cancer therapy (22, 23). Therefore, elucidating the relationship between FRG expression and HCC prognosis and identifying ferroptosis-specific therapeutic targets in HCC is of great clinical significance in enhancing the efficacy of tumor treatment and improving the prognosis of HCC patients.

In this study, we systematically investigated the expression landscape of FRGs in HCC using the TCGA cohort and identified 43 FRGs that were differentially expressed between tumor and peri-tumor tissues. Functional enrichment analysis revealed that these genes were mainly involved in ferroptosis-related pathways, including metabolic and oxidative processes. Next, we observed that 11 of the 43 FRGs were statistically significantly associated with both OS and RFS, suggesting that FRGs affect the prognosis of patients with HCC. Epigenetic alterations have a profound impact on cancer genesis, development, and progression, and aberrant methylation changes frequently occur in tumors (24). Among the dysregulated DNAm driver genes, some may facilitate malignant transformation through oncogene overexpression, thereby creating a new balance in the TME and potentially serving as diagnostic and prognostic biomarkers (25). Therefore, the UALCAN database was used to explore the DNA methylation patterns of 11 prognosis-related FRGs that were aberrantly expressed in HCC. Four methylation-related ferroptosis genes (G6PD, HELLS, RRM2, and STMN1) were identified. Although these four methylation-related FRGs have previously been reported to be dysregulated in cancer or other diseases, their biological roles in HCC have not yet been thoroughly elucidated.

For instance, glucose-6-phosphate dehydrogenase (G6PD) plays an essential role in glucose metabolism by maintaining redox homeostasis and reductive biosynthesis in cells (26). The aberrant activation of G6PD contributes to enhanced cell proliferation and adaptation in multiple cancer types (26). Moreover, lymphocyte-specific HELLS, a SWI2/SNF2 chromatin remodeling enzyme, is upregulated in pancreatic cancer tissues and correlates with an advanced clinical stage and poor prognosis (27). However, the regulatory mechanisms for HCC remain unclear. Ribonucleotide reductase subunit M2 (RRM2) has been extensively reported to be involved in the progression of various tumors such as renal cell cancer (28) and lung cancer (29). However, the role of RRM2 in HCC remains to be elucidated. Stathmin 1 (STMN1), a gene encoding a cytoplasmic phosphoprotein, plays an important role in cell cycle progression, mitosis, and signal transduction (30).

Next, the frequency of genetic alterations in the four methylation-driven FRGs was investigated in HCC. The four FRGs mostly showed genetic alterations, such as gene amplification, and genetic alterations in the four FRGs were significantly associated with worse OS (P = 1.112e-3). Additionally, G6PD, HELLS, RRM2, and STMN1 were positively associated with higher tumor stage, grade, age, sex, and ethnicity, suggesting an influential contribution to the pathogenesis of HCC. We further validated the transcriptome and protein expression levels of the four central FRGs using our cohort of HCC patients and downloaded datasets, and the results demonstrated that the transcriptome and proteome expression levels were consistent. This also strengthens the reliability of the screened genes. To evaluate the classification performance of the G6PD, HELLS, RRM2, and STMN1 signatures, we constructed four FRGs prediction models using TCGA database. ROC curves showed that all four genes could be used as biomarkers to sensitively and accurately distinguish tumors from normal liver tissue. The prognostic value of these four FRGs signatures was further validated using the GSE14520 dataset. All four FGRs appear to be promising therapeutic targets and prognostic predictors. To further reveal the biological functions of the hub genes, we conducted GSEA for the four hub FGRs. The results indicated that some HALLMARK pathways related to tumorigenesis and cell cycle regulation, such as “G2M checkpoint,” “mitotic spindle,” “E2F targets,” and “DNA repair” were enriched in the high expression group of these pivotal FGRs, suggesting that their contribution to HCC proliferation progression. This indicated that our predictive model has promising clinical application value and could provide potential insights for the development of new targeted therapies.

Tumor-infiltrating immune cells exert antitumor immune functions by releasing cytokines that promote iron death in tumor cells (31). Our study showed that the expression of the four FRGs was associated with the majority of infiltrating immune cells in HCC tissue specimens, suggesting that ferroptosis may have a significant effect on hepatic tumor immunity. Similarly, in recent years, immunotherapy using immune checkpoint inhibitors (ICIs) has shown unprecedented breakthroughs in the treatment of various types of malignancies, as the immune checkpoint mechanism has been intensively studied (32). For instance, PD-1, LAG-3, TIM3, CTLA-4, and TIGIT antibodies have shown favorable results in relevant clinical trials (33, 34). However, the objective response rate and survival benefit of ICIs for numerous tumors remain low, in part due to the lack of tumor-infiltrating lymphocytes (35). Transforming these immune “cold” tumors into “hot” tumors that respond to ICIs is a hot topic in current research. Recently, ferroptosis was found to have a profound effect on the tumor immune response. Specifically, ferroptosis exhibits a synergistic antitumor immune response and may also have a suppressive effect on the antitumor immune response (31). In this study, we investigated the relationship between four FRGs and immune checkpoints. The results showed that the high expression of the four FRGs was strongly associated with PDCD1, LAG3, HAVCR2, CTLA-4, and TIGIT in HCC, suggesting that targeting ferroptosis may enhance the efficacy of immunotherapy in HCC.

## Conclusion

Collectively, this study determined novel predictive models for four FRGs using multiple cohort datasets and bioinformatic analysis. The expression patterns of the four FRGs that were upregulated in HCC tissues may be associated with hypomethylation. Furthermore, our current findings also revealed that the four FRGs may influence HCC development by increasing tumor immune cell infiltration and immune checkpoint expression, which provides new insights into targeting or immunotherapy ferroptosis-related targets. However, further studies are needed to demonstrate their clinical value.

## Data availability statement

The data analyzed in this study can be found on the TCGA database (https://portal.gdc.cancer.gov/), GEO database (https://www.ncbi.nlm.nih.gov/gds), and ICGC database (https://dcc.icgc.org/).

## Author contributions

YZ, LC, and HR designed and drafted the manuscript; HR, CZ, and HL collected related data and conducted the bioinformatics analysis; HX, LC, and QG revised the image and manuscript. All authors contributed to the article and approved the submitted version.

## Funding

This work was supported by the Beijing Medical Award (grant number YXJL-2021-0268-0322) and the Haiyan Fund of Harbin Medical University Cancer Hospital (No. JJMS2021-22 and JJQN2019-17).

## Conflict of interest

The authors declare that the research was conducted in the absence of any commercial or financial relationships that could be construed as a potential conflict of interest.

## Publisher’s note

All claims expressed in this article are solely those of the authors and do not necessarily represent those of their affiliated organizations, or those of the publisher, the editors and the reviewers. Any product that may be evaluated in this article, or claim that may be made by its manufacturer, is not guaranteed or endorsed by the publisher.

## Abbreviations

FRGs, ferroptosis-related genes; HCC, Hepatocellular carcinoma; FDR, False discovery rate; ROC, Receiver Operator Characteristic; OS, overall survival; RFS, relapse-free survival; DFS, disease-free survival; CNV, gene copy number variation; DEGs, Differentially expressed genes; GO, Gene Ontology; KEGG, Kyoto Encyclopedia of Genes and Genomes; GSEA: Gene Set Enrichment Analysis; TME, The Tumor Microenvironment; ICIs, Immune Checkpoint Inhibitors.
