# Supplementary material for: Development and validation of four ferroptosis-related gene signatures and their correlations with immune implication in hepatocellular carcinoma
Source: Front Immunol. 2022 Oct 11;13:1028054. doi: 10.3389/fimmu.2022.1028054 (PMC9592986; doi:10.3389/fimmu.2022.1028054)
Supplement: Supplementary file 2 [file Table_1.docx]

| **Gene name** |  |  | **Sequence of primer** |
| --- | --- | --- | --- |
| G6PD |  |  | F: CGAGGCCGTCACCAAGAAC  R: GTAGTGGTCGATGCGGTAGA |
| HELLS |  |  | F: AGAAGGCATGGAATGGCTTAGG  R: GCCACAGACAAGAAAAGGTCC |
| RRM2 |  |  | F: GTGGAGCGATTTAGCCAAGAA  R: CACAAGGCATCGTTTCAATGG |
| STMN1 |  |  | F: TCAGCCCTCGGTCAAAAGAAT  R: TTCTCGTGCTCTCGTTTCTCA |

**Table S1.** **mRNA PCR primer**.
